# Genome-scale analyses of transcriptional start sites in *Mycobacterium marinum* under normoxic and hypoxic conditions

**DOI:** 10.1186/s12864-021-07572-8

**Published:** 2021-04-06

**Authors:** Shaojia Huang, Wei Zhou, Wei Tang, Yong Zhang, Yangbo Hu, Shiyun Chen

**Affiliations:** 1 CAS Key Laboratory of Special Pathogens and Biosafety, Wuhan Institute of Virology, Center for Biosafety Mega-Science, Chinese Academy of Sciences, Wuhan, 430071 China; 2 University of Chinese Academy of Sciences, Beijing, 100049 China

## Abstract

**Background:**

Hypoxic stress plays a critical role in the persistence of *Mycobacterium tuberculosis* (Mtb) infection, but the mechanisms underlying this adaptive response remain ill defined.

**Material and methods:**

In this study, using *M. marinum* as a surrogate, we analyzed hypoxic responses at the transcriptional level by Cappable-seq and regular RNA-seq analyses.

**Results:**

A total of 6808 transcriptional start sites (TSSs) were identified under normoxic and hypoxic conditions. Among these TSSs, 1112 were upregulated and 1265 were downregulated in response to hypoxic stress. Using SigE-recognized consensus sequence, we identified 59 SigE-dependent promoters and all were upregulated under hypoxic stress, suggesting an important role for SigE in this process. We also compared the performance of Cappable-seq and regular RNA-seq using the same RNA samples collected from normoxic and hypoxic conditions, and confirmed that Cappable-seq is a valuable approach for global transcriptional regulation analyses.

**Conclusions:**

Our results provide insights and information for further characterization of responses to hypoxia in mycobacteria, and prove that Cappable-seq is a valuable approach for global transcriptional studies in mycobacteria.

**Supplementary Information:**

The online version contains supplementary material available at 10.1186/s12864-021-07572-8.

## Introduction

*Mycobacterium tuberculosis* (Mtb), the etiologic agent of tuberculosis (TB), is one of the most successful bacterial pathogens of humans. It is estimated that one-third of the world’s population is infected by Mtb, of whom 90% will develop latent TB infection (LTBI) [1], presenting a heavy burden for TB prevention. Through inhalation of aerosol droplets containing bacilli, Mtb reaches lung airways and is engulfed by alveolar macrophages, which then recruit mononuclear cells and T lymphocytes to form granulomas [2]. In both macrophages and granulomas, Mtb encounters hypoxic stress [3, 4], which has been shown to be important for inducing the transition to dormancy, a nonreplicating and drug-resistant state [1, 5].

Several in vitro models have been established to investigate how Mtb adapts to hypoxic stress, such as the Wayne model [6] and the defined hypoxia model [7, 8]. In the Wayne model, bacterial cells are grown in sealed and stirred tubes with a defined headspace-to-culture ratio, allowing the gradual exhaustion of oxygen [6]. Compared with the defined hypoxia model, which requires a constant flow of low oxygen gas over the culture to maintain hypoxia [7, 8], the Wayne model is relatively simple to set up in the laboratory [5, 6, 9].

It has been reported that the two-component response regulator DosR is critical for the initial hypoxic response [8, 10], and hundreds of genes are subsequently upregulated independent of DosR, defining the enduring hypoxic response (EHR) [9]. The regulatory networks of Mtb during hypoxia and re-aeration have been reconstructed based on ChIP-Seq analyses [11]. These studies suggest that transcriptional regulators play essential roles in hypoxic responses. In addition to transcriptional regulators, sigma factors have also been known to regulate gene transcription in stress responses. Sigma factor (σ) first reconstitutes with the RNA polymerase (RNAP) core enzyme to form a holoenzyme, which can recognize specific promoter sequences in the transcription initiation process [12]. Bacterial promoters of the σ^70^ family contain − 10 and − 35 elements located upstream of transcriptional start sites (TSSs) [13]. Therefore, global identification of TSSs would provide valuable information for promoter characterization. The Mtb genome encodes 13 sigma factors, most of which are critical for survival under stress conditions [14–18]. Among them, *sigB* and *sigE* have been shown to be upregulated under hypoxic stress [19], although their functions in hypoxic responses have not been completely characterized.

In recent studies, different methods have been developed for the global analysis of TSSs in bacteria, such as differential RNA-seq (dRNA-seq) [20] and Cappable-seq [21]. In prokaryotes, primary transcripts reflecting TSSs constitute less than 5% of the total RNA; the vast majority are processed ribosomal RNAs. Taking advantage of the distinctive 5′ triphosphate ends of primary transcripts, dRNA-seq and Cappable-seq use different strategies to enrich for primary transcripts. dRNA-seq relies on 5′ monophosphate-dependent terminator exonuclease (TEX) to deplete processed transcripts, thus enhancing the relative content of primary transcripts [20]. In Cappable-seq, vaccinia capping enzyme (VCE) is used to label 5′ triphosphorylated RNA with a biotin derivative, and then the primary transcripts are captured on streptavidin beads [21]. Global characterizations of TSSs in different mycobacterial species have also been documented [22–26], but the influences of different stresses on the strength of these TSSs in mycobacteria are not well defined.

*M. marinum* has been used as a surrogate for Mtb to overcome its slow-growing nature and to avoid the requirement of a biosafety level 3 (BSL-3) facility. More importantly, *M. marinum* also forms granulomas during zebrafish infection [27, 28]. In this study, we characterized the transcriptional regulation of *M. marinum* under normoxic and hypoxic conditions by quantifying the strength of TSSs using Cappable-seq [21], which provides insights and information for further characterization of hypoxic responses in mycobacteria.

## Results

### Hypoxic treatment of *M. marinum*

**Fig. 1:**
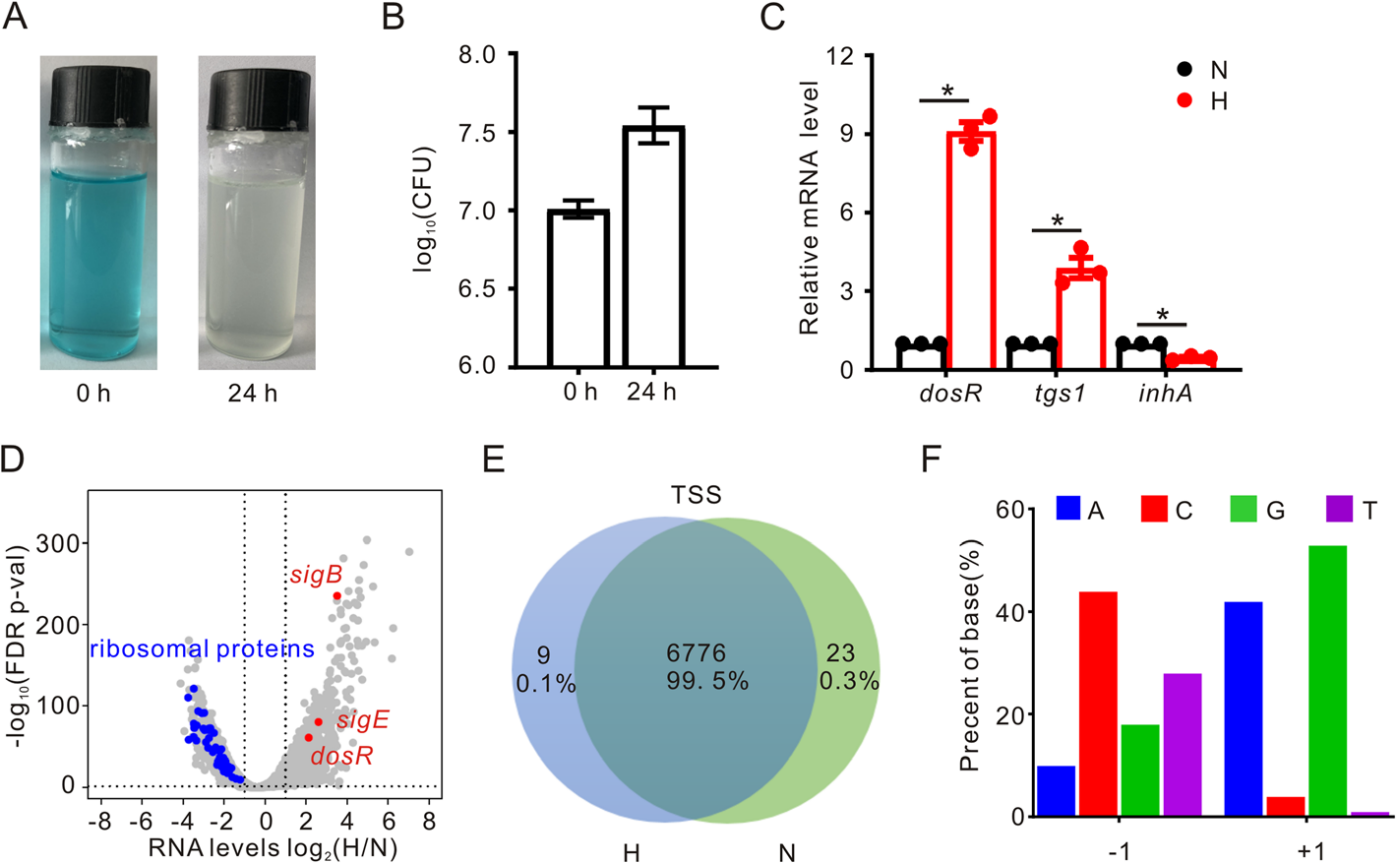
Global mapping of TSSs under normoxic (N) and hypoxic (H) conditions. **a** *M. marinum* cultures prior to (0 h) and 24 h after hypoxic incubation. **b** The CFU numbers of *M. marinum* prior to and 24 h after hypoxic incubation. Data are shown as the mean ± SEM values of three experiments performed in triplicate. **c** Transcriptional levels of *dosR*, *tgs1* and *inhA* genes under normoxic and hypoxic conditions. Relative transcriptional levels of these genes under normoxic conditions were normalized to 1. Data are shown as the mean ± SEM values of biological triplicates performed in duplicate. *, *p* < 0.05. **d** Volcano plot showing the differential expression of global mRNA as a ratio of expression under hypoxic (H) and normoxic (N) conditions. The blue dots are downregulated genes encoding ribosomal proteins, and the red dots are upregulated *dosR*, *sigE* and *sigB* genes. Data are shown as the mean values of biological triplicates. FDR-adjusted *p*-values were obtained using DESeq2. **e** Venn diagram showing the numbers and percentages of TSSs identified under normoxia and hypoxia. **f** Percentages of base preference at the − 1 and + 1 (relative to TSSs) positions of 6808 TSSs identified under normoxia and hypoxia

To test the hypoxic response of *M. marinum*, we applied the Wayne low-oxygen model to mimic hypoxic conditions [3, 6]. In this model, an oxygen-responsive dye, methylene blue, was added to the medium to indicate the depletion of oxygen. As shown in Fig. 1a, the dye changed from blue to colorless when incubated at 32 °C for 24 h. *M. marinum* cell cultures with and without hypoxic treatment were collected. Colony forming units (CFU) were increased approximately 3-fold after 24 h under oxygen limitation (Fig. 1b), suggesting that the cells successfully coped with hypoxic stress. As positive controls, the induction of the dormancy regulator *dosR* and one of its activated genes, *tgs1* [10], under hypoxic conditions was confirmed using qRT-PCR analysis. Consistent with a previous report [11], the expression level of *inhA*, which is known to be involved in mycolic acid biosynthesis [29], was decreased (Fig. 1c).

To further confirm the hypoxic responses of *M. marinum* in the Wayne low oxygen model, we performed regular RNA-seq and found a total of 5112 and 5362 coding sequences (CDSs) with fragments per kilobase per million mapped fragments (FPKM) values of ten or more under normoxic and hypoxic conditions, respectively (Table S1). We observed numerous differentially expressed genes, among which 1087 were upregulated and 1791 were downregulated in response to hypoxic stress (Fig. 1d, Table S1). Several transcriptional regulators, such as *dosR*, *sigE,* and *sigB*, were upregulated, whereas most ribosomal genes were downregulated (Fig. 1d). These results confirm that *M. marinum* cells experienced hypoxic stress in our Wayne model.

### Global mapping of TSSs in *M. marinum* under normoxic and hypoxic conditions

To facilitate a detailed regulatory analysis of the transcriptional regulation of *M. marinum* under hypoxic stress, we next identified TSSs at the genome-scale under normoxic and hypoxic conditions by Cappable-seq [21]. On average, over one million reads were uniquely mapped to the *M. marinum* genome outside of the ribosomal and tRNA regions. By analyzing the first nucleotide at the 5′ end of the mapped reads, we identified a total of 6808 TSSs (Fig. 1e, Table S2). Consistent with other reports [21, 22], purine bases were enriched at the + 1 position (A + G, 95.8%), and pyrimidine bases were enriched at the − 1 position (C + T, 72.8%) (Fig. 1f). Moreover, 2046 out of 6808 of TSSs identified in our study matched completely and 636 out of 6808 TSSs matched with 1 bp or 2 bp difference compared with data from a previous dRNA-seq analysis [24] (Table S2). These data corroborate that Cappable-seq is a valuable approach for TSS identification.

### Characterization of *M. marinum* promoters

**Fig. 2:**
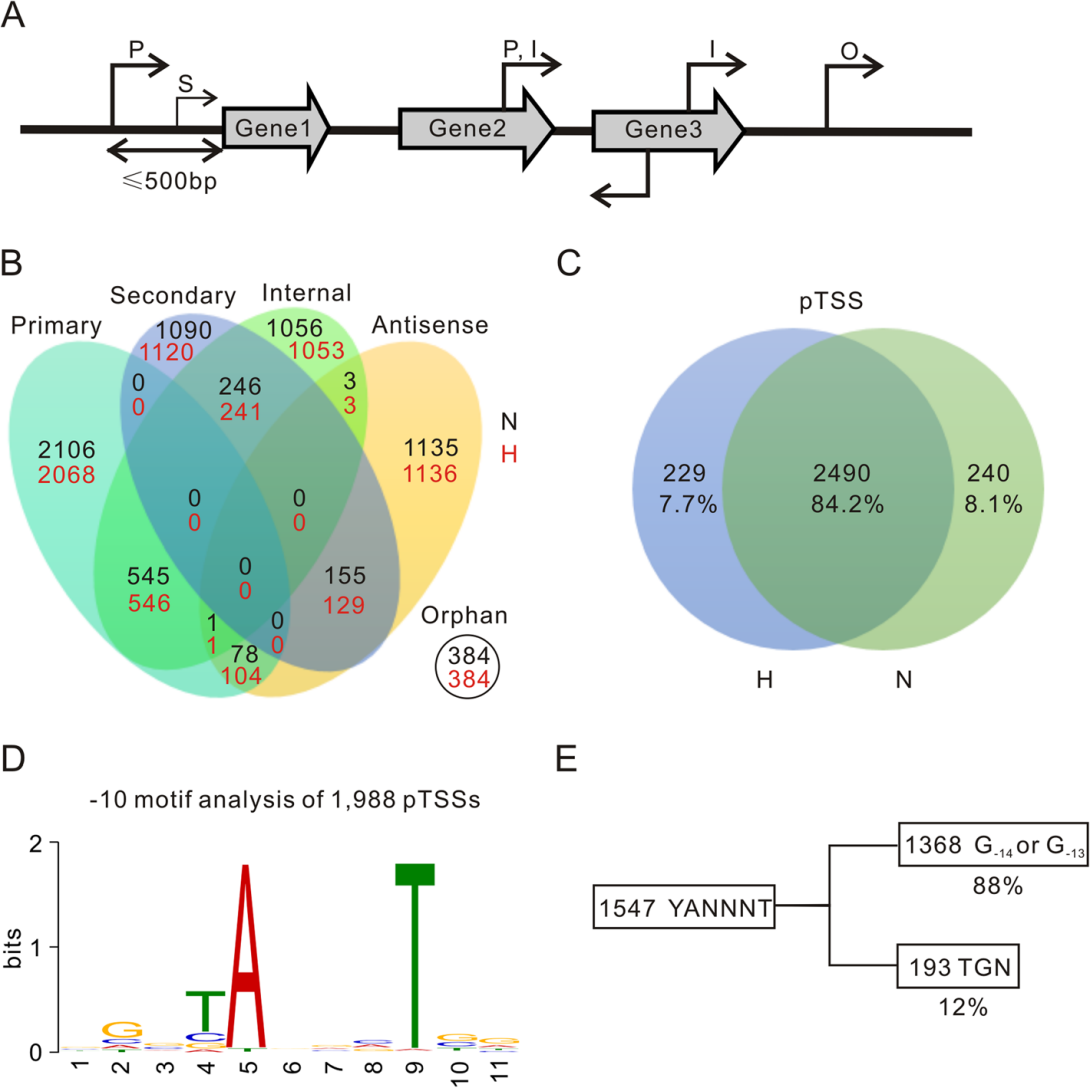
Classifications of TSSs and the features of promoters in *M. marinum.* **a** Classification scheme for TSSs based on genome position and TSS strength: primary (P), secondary (S), internal (I), antisense (A) and orphan (O). **b** Venn diagrams showing classifications of TSSs identified under normoxic (N, black color) and hypoxic (H, red color) conditions. A TSS could be classified into more than one category. **c** Venn diagram showing the numbers and percentages of pTSSs identified under normoxia and hypoxia. **d** Consensus for − 10 motif of 1988 promoters upstream of pTSSs expressed under both normoxia and hypoxia generated by MEME [30, 31]. Regions containing 12 bp (− 5 to − 16 relative to TSSs) were used for analysis. **e** Numbers and percentages of G_− 14_ or G_− 13_ and TGN preceding the − 10 YANNNT motif

To gain insight into the transcriptional organization of *M. marinum*, we classified TSSs into five categories according to genome position and TSS strength as previously reported [20]: primary TSS (pTSS), secondary TSS (sTSS), internal TSS (iTSS), antisense TSSs (aTSS) and orphan TSS (oTSS) (Fig. 2a and b). A total of 2959 pTSSs were identified, which covered more than half of the 5557 genes in *M. marinum*. Among them, 2490 pTSSs were found under both normoxic and hypoxic conditions, and 229 pTSS were identified only under hypoxic conditions (Fig. 2c), indicating stress-induced transcription. Moreover, a total of 1853 iTSSs, 1375 aTSSs, 385 oTSSs, and 1490 sTSSs were identified. Notably, some aTSSs and oTSSs may initiate the transcription of small noncoding RNAs or represent the products of spurious transcription initiation, which requires further investigation.

To characterize the common features of *M. marinum* promoters, we used 50 bp sequences upstream of 2490 pTSSs to search for conserved motifs using MEME [30, 31]. A − 10 motif was found upstream of 80% (1988/2490) of the pTSSs, among which YANNNT (1547/2490, 62%) was the most commonly identified sequence (Fig. 2d). We could not detect a consensus − 35 motif, which is consistent with previous reports in Mtb and *M. smegmatis* [22, 25, 26]. Furthermore, T_−15_G_− 14_N (TGN) has been described to form an extended − 10 consensus in several bacterial promoters, and G_− 14_ or G_− 13_ together with the − 10 consensus has been characterized as a minimal promoter type in mycobacteria [32, 33]. We analyzed the frequency of TGN, G_− 14_ or G_− 13_ upstream of the − 10 motif, and found that only 12% (193/1547) of YANNNT motifs were preceded by TGN and 88% (1368/1547) of YANNNT motifs were preceded by G_− 14_ or G_− 13_ (Fig. 2e). It is possible that the relatively high percentage of G_− 14_ or G_− 13_ motif compensates for the deficiency of the − 35 motif in mycobacteria [33, 34].

### Widespread leaderless transcripts in *M. marinum*

**Fig. 3:**
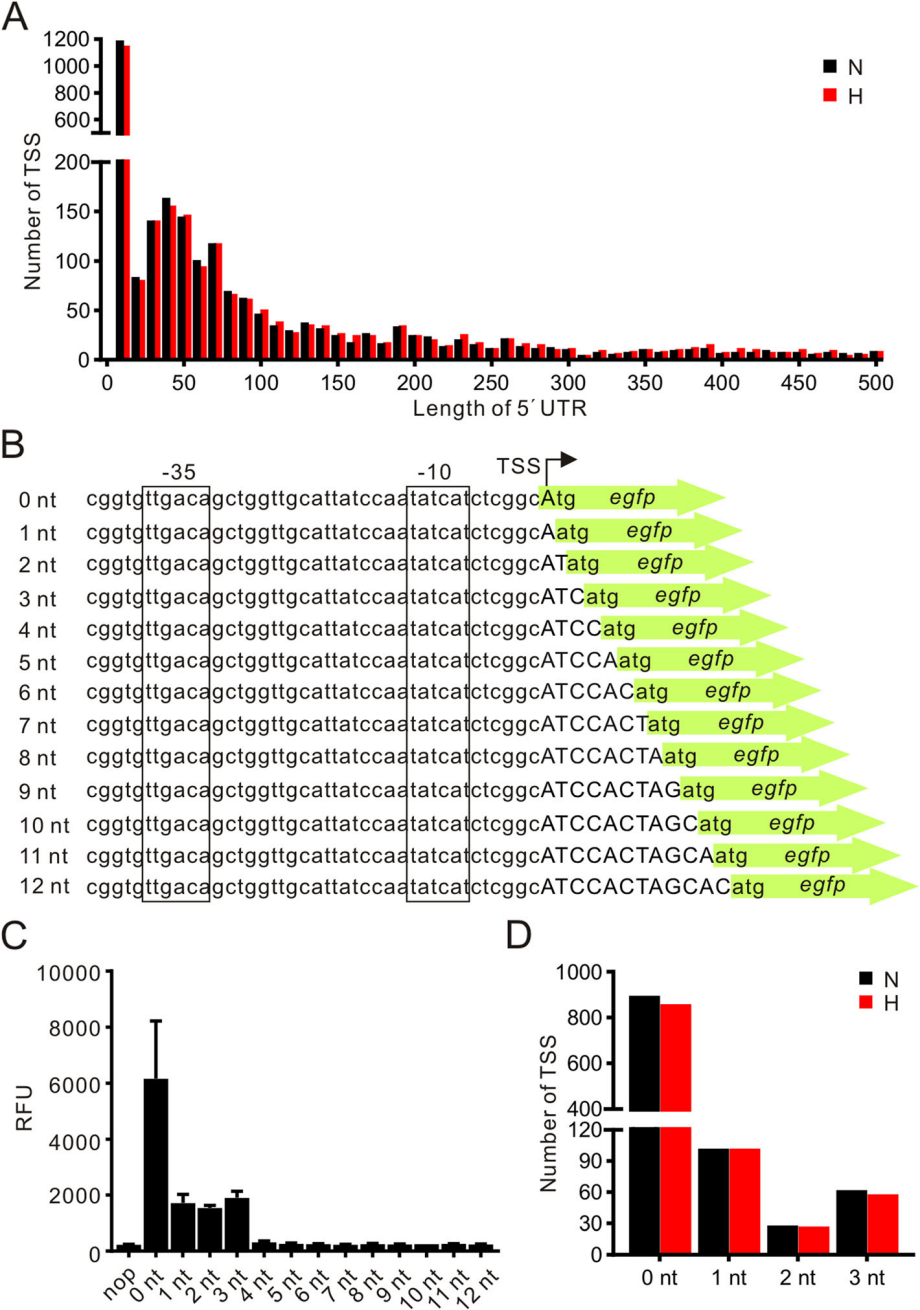
Leaderless transcripts are common in *M. marinum.* **a** Distribution of 5′ UTR length (10 nt bins) of mRNAs with a primary TSS. Black and red bars indicate mRNAs associated with 2730 pTSSs and 2719 pTSSs identified under normoxia (N) and hypoxia (H), respectively. **b** Sequences of promoters and 5′ UTRs of *egfp* transcribed with different lengths of 5′ UTR. **c** Relative fluorescence units (RFU) of *egfp* transcribed with of 5′ UTRs of different lengths, from 0 nt to 12 nt. Data are shown as the mean ± SD values from two independent colonies. “nop” indicates the background value for measuring RFU. **d** Numbers of pTSS initiating the transcription of functional leaderless transcripts under normoxic (N) and hypoxic (H) conditions

Although the mechanism of translation from leaderless mRNA has not been completely characterized, leaderless transcripts have been reported as a prominent feature in both Mtb and *M. smegmatis* [22, 25]. To determine whether this feature exists in *M. marinum*, we analyzed the length of the 5′ untranslated region (5′ UTR) associated with pTSSs. A large number of 5′ UTR sequences are shorter than 10 nt, suggesting a possible richness of leaderless RNAs in *M. marinum* (Fig. 3a). To characterize the relationship between 5′ UTR length and translational efficiency, we tested the expression of eGFP with 5′ UTR lengths ranging from 0 to 12 nt (Fig. 3b). Interestingly, we found that transcripts with a 5′ UTR less than 4 nt could be efficiently translated (Fig. 3c), suggesting that transcripts with a 0–3 nt 5′ UTR could be defined as functional leaderless transcripts. Based on these analyses, we revealed that a total of 1288 TSSs initiate leaderless transcripts in *M. marinum* (Table S2), and 991 of these TSSs were reported [24]. Interestingly, 39.8% (1088/2730) and 38.4% (1045/2719) of pTSSs initiate functional leaderless transcripts under normoxia and hypoxia respectively (Fig. 3d).

### Transcriptional regulation in response to hypoxic stress

**Fig. 4:**
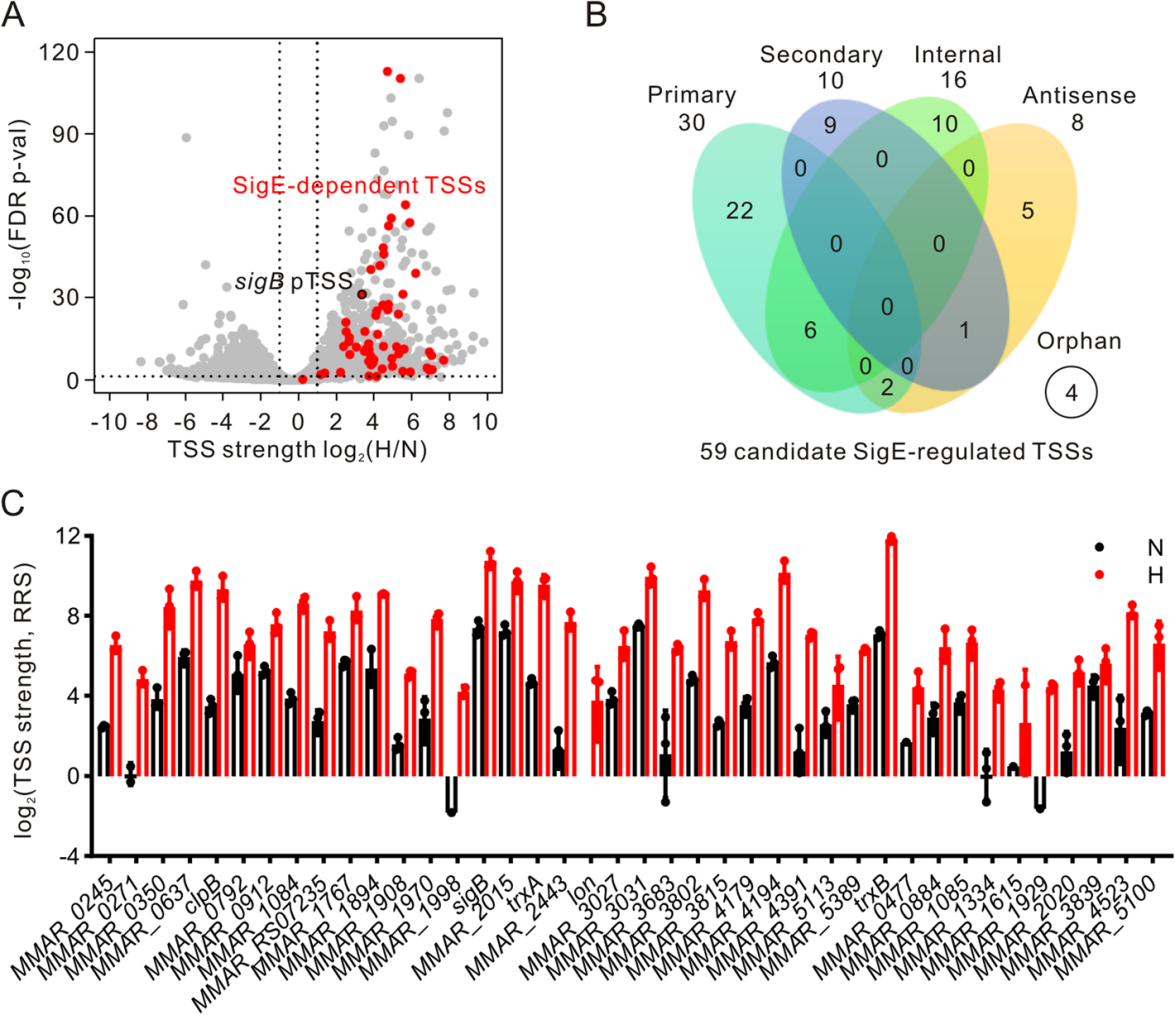
Global characterization of transcriptional regulation in response to hypoxic stress. **a** Volcano plot showing fold-changes of TSS strength as a ratio of hypoxic (H) and normoxic (N) incubation. The red dots are 59 SigE-dependent TSSs, including the pTSS of *sigB* (marked). Data are shown as the mean values of biological triplicates. FDR-adjusted p-values were obtained using DESeq2. **b** Classification of 59 SigE-dependent TSSs. **c** TSS strength of SigE-dependent pTSSs and sTSSs under normoxia (N) and hypoxia (H). The first 30 genes are associated with pTSSs and the last 10 genes are associated with sTSSs. Data are shown as the mean ± SD values of biological triplicates

By quantifying the strength of TSSs under normoxic and hypoxic conditions, we analyzed the global transcriptional regulation of *M. marinum* in hypoxic responses. Of the 6808 TSSs identified under either condition, 9 TSSs were specifically detected under hypoxic conditions and 23 TSSs were specifically detected under normoxic conditions (Fig. 1e, Table S3). In addition, 1103 TSSs were significantly upregulated and 1242 TSSs were significantly downregulated in response to hypoxic stress (fold change ≥2, FDR *p*-value < 0.05; Fig. 4a) (Table S2). Table 1 shows the top 15 upregulated and downregulated pTSS meeting the criterion of a relative read score (RRS) of 20 or more in all three replicates. Among them, the pTSS of *sigB* was in the top 15 upregulated pTSSs, which is consistent with our observation in regular RNA-seq data that the mRNA level of *sigB* was increased under hypoxic conditions. For the 15 most downregulated pTSSs, 2 genes downstream of these pTSSs encoded synthetases, which is consistent with the low metabolic activity observed under hypoxic conditions.

**Table 1:** Top 15 pTSSs up- or downregulated under hypoxic stress

Position	Associated gene	FC (H/N)	Product
**upregulated**			
2,741,615, +	*trxB1*	30.3	thioredoxin
5,166,688, +	*kgd*	23.1	multifunctional oxoglutarate decarboxylase
5,928,771, +	*MMAR_4874*	23.1	transcriptional regulator
19,429, +^a^	*MMAR_p23*	23.0	chromosome partitioning protein
4,722,828, −	*porB*	22.6	2-oxoacid ferredoxin oxidoreductase subunit beta
3,034,066, −	*MMAR_2491*	18.2	zinc ribbon domain-containing protein
5,952,502, +	*MMAR_4896*	16.7	hypothetical protein
2,306,716, +	*infB*	11.5	translation initiation factor IF-2
1,233,867, +	*MMAR_1007*	10.4	transcriptional regulator
2,424,872, −	*sigB*	10.4	RNA polymerase sigma factor
4,231,315, −	*MMAR_3428*	10.1	hypothetical protein
1,233,815, −	*mmpS5*	9.6	membrane protein
3,478,075, +	*MMAR_2872*	8.4	multidrug-transport integral membrane protein
5,945,575, −	*purL*	7.9	phosphoribosylformylglycinamidine synthase subunit
6,373,356, +	*MMAR_5280*	7.3	dihydrodipicolinate reductase
**downregulated**			
3,623,295, −	*MMAR_2999*	0.016	OsmC family peroxiredoxin
2,115,027, +	*fadD29*	0.054	acyl-CoA synthetase
5,624,834, +	*mntH*	0.072	divalent metal cation transporter
3,801,485, −	*MMAR_3116*	0.083	undecaprenyl-diphosphate phosphatase
2,817,295, +	*fadD25*	0.084	acyl-CoA synthetase
2,816,979, −	*pks5*	0.095	probable polyketide synthase
2,686,224, +	*MMAR_2235*	0.095	PE-PPE domain-containing protein
2,838,256, −	*MMAR_2352*	0.097	GAP family protein
2,966,269, +	*pheT*	0.103	phenylalanine-tRNA ligase subunit beta
1,541,144, +	*fadE25*	0.117	acyl-CoA dehydrogenase
5,174,763, −	*mdh*	0.124	malate dehydrogenase
572,814, −	*MMAR_5555*	0.130	hypothetical protein
3,298,910, +	*gcvH*	0.132	glycine cleavage system protein H
4,257,552, +	*MMAR_3453*	0.136	pyridoxal phosphate-dependent aminotransferase
1,249,089, +	*MMAR_1023*	0.137	mycofactocin biosynthesis peptidyl-dipeptidase

^a^ pTSS locates in plasmid (NC_010604.1)

Altering the ratio of alternative sigma factor with primary sigma factor could regulate the transcription of many genes and is a common strategy used by bacteria to survive in diverse harsh conditions [35]. Consistent with previous reports that the expression of *sigE* and *sigB* was induced in Mtb under hypoxic stress [19], we observed that the pTSS of *sigB* was one of the most upregulated TSSs under hypoxic conditions in *M. marinum* (Table 1). As the promoter of the *sigB* gene was activated by SigE [36], we further analyzed whether other SigE-dependent promoters were activated under hypoxia. We took advantage of the reported SigE-dependent promoter consensus GGGAACY-N_16–17_-CGTT [37] to search for promoters recognized by SigE. In total, we obtained 59 TSSs of different categories (Fig. 4b) that were elevated in response to hypoxia (Table S2, Fig. 4a, c).

### Comparison of Cappable-seq and regular RNA-seq in global transcriptional regulation analyses

**Fig. 5:**
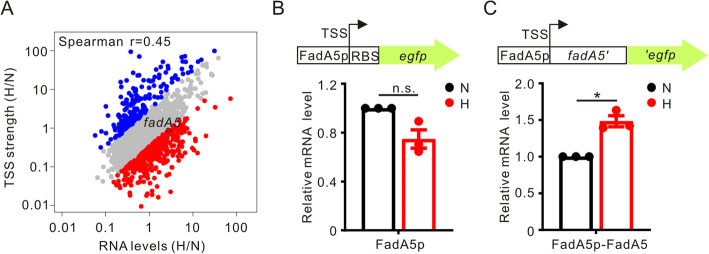
Comparison of regular RNA-seq and Cappable-seq in global transcriptional analyses. **a** Correlation between fold-changes of RNA levels and TSS strength under hypoxia (H) compared with normoxia (N) from regular RNA-seq and Cappable-seq data. The graph containing 2387 pTSSs expressed under both normoxia and hypoxia and the expression of downstream genes of these pTSSs were detected by regular RNA-seq. The red and blue dots indicate 393 upregulated and 175 downregulated genes that probably subjected to posttranscriptional regulation. The *fad5A* gene was chosen for verification using qRT-PCR. **b** and **c** Transcriptional (**b**) and translational (**c**) fusions of *fadA5* to *egfp.* The primers for qRT-PCR were designed according to the *egfp* sequence. Relative transcriptional levels under normoxic conditions were normalized to 1. Data are shown as the mean ± SEM values from biological triplicates performed in duplicate. *, *p* < 0.05; n.s., not significant

We next compared the data in Cappable-seq with our data in regular RNA-seq to confirm the efficiency of Cappable-seq in the global analysis of transcriptional regulation. Among 2490 pTSSs expressed under both normoxia and hypoxia, 2387 corresponded to genes whose transcripts were detected in regular RNA-seq. We compared the fold-changes of RNA levels with TSS strength, and a moderate correlation was observed (r = 0.45; Fig. 5a). As expected, most of the genes showed similar patterns of regulation in both methods (Fig. 5a). The small differences observed may be due to posttranscriptional regulation or other unknown factors. Together, these analyses indicate that Cappable-seq is a valuable method for transcriptional analysis.

Inconsistencies between Cappable-seq and regular RNA-seq may represent genes that are subject to posttranscriptional regulation. Therefore, we selected genes with a ratio of fold-change (H/N) of RNA levels to fold-change (H/N) of TSS strength greater than 3 or less than 0.33. Interestingly, a total of 568 genes (Fig. 5a), including 393 upregulated genes (ratio > 3) and 175 downregulated genes (ratio < 0.33), were suggested to be regulated at the posttranscriptional level under hypoxic conditions. Considering its function and homology to Mtb, we chose one of upregulated gene, *fadA5*, to validate posttranscriptional regulation. FadA5 is involved in fatty acid metabolism and is a leaderless transcript in both *M. marinum* and Mtb [22]. We respectively constructed the *fadA5* promoter and translational fusions to *egfp* using the integrative vector pMV306 [38]. *M. marinum* carrying *egfp* fusion plasmids was grown under normoxia and hypoxia. In qRT-PCR assays to detect *egfp* mRNA levels, we did not observe a significant difference in *fadA5* promoter activity in response to hypoxic stress (Fig. 5b), but the level of the translational fusion *fadA5*::*egfp* increased 1.5-fold in response to hypoxic stress (Fig. 5c). These results suggested that the stability of *fadA5* mRNA might be increased under hypoxic conditions and supported our proposal that combining regular RNA-seq with Cappable-seq could provide a means to globally investigate posttranscriptional regulation.

## Discussion

*M. marinum* has been used as a surrogate for Mtb [27, 39, 40]. Compared with *M. smegmatis*, *M. marinum* is more closely genetically related to Mtb, and an *M. marinum*-zebrafish infection model has been established to study latent infection [28, 41]. Recently, genome-scale mapping of TSSs in *M. smegmatis* during aerobic growth and under hypoxia has been reported [26], but how hypoxic stress affects the strength of TSSs in *M. marinum* is still unclear. In this study, we applied Cappable-seq to quantifiably identify the changes in TSSs in *M. marinum* and globally analyze transcriptional regulation in response to hypoxic stress. We totally identified 6808 TSSs. Among them the strength of 1112 TSSs were upregulated and 1265 TSSs were downregulated under hypoxic stress. Moreover, comparing the performance of Cappable-seq and regular RNA-seq, we demonstrated that Cappable-seq is a valuable method for global transcriptional analysis.

Developed from regular RNA-seq, several other modified methods have been performed to study specific characteristics of the transcriptome. dRNA-seq and term-seq have been used to identify the 5′ and 3′ boundaries of transcripts, respectively, at genome-scale [20, 42]. Cappable-seq is a recently developed high-throughput method to determine TSSs, which has been applied in *E. coli*, *Streptococcus pneumoniae* and *Photorhabdus luminescens* [21, 43, 44]. In this study, nearly half of TSSs characterized by Cappable-seq were also identified by dRNA-seq analysis [24], suggesting that these methods are highly reliable and reproducible. In addition, Cappable-seq was reported to have higher sensitivity than dRNA-seq, and thus could reveal novel TSSs that are weakly expressed in *E. coli* [21]. In *M. marinum*, we also found a higher number of TSSs in Cappable-seq specific TSS that were not identified in dRNA-seq [24].

Recently, the transcriptional regulation of *M. marinum* at different time points of resuscitation from hypoxia-induced dormancy has been characterized [45], which revealed that 8 transcription factors, i.e., *MMAR_0923*, *MMAR_1394*, *MMAR_1653*, *MMAR_4219*, *MMAR_4852* (*kmtR*), *MMAR_4874* (*cosR*), *MMAR_5170* (*whiB4*) and *MMAR_5405* (*ethR*), were significantly downregulated upon resuscitation from dormancy. In our study, 4 of these transcription factors (*MMAR_0923*, *MMAR_4219*, *kmtR* and *cosR*) were highly upregulated in regular RNA-seq (Table S1) and 5 of these genes (*MMAR_0923*, *MMAR_1394*, *MMAR_1653*, *cosR* and *kmtR*) contained TSSs that were upregulated under hypoxic conditions (Table S2), confirming the importance of these transcription factors in coping with hypoxic stress. Importantly, our Cappable-seq data provided the sequences of these regulated promoters, which could facilitate research on target identifications as well as functional analyses of these transcription factors.

Several studies have performed transcriptomic analyses of Mtb under hypoxic conditions [9, 11, 46]. The expression of transcription factors, such as *Rv0081*, *Rv1353c* and *cosR* (*Rv0967*), was reported to be induced in response to hypoxia [9, 11, 46]. In our Cappable-seq data, pTSSs of these homologous genes (*MMAR_1653*, *MMAR_4019* and *MMAR_4874*) were identified and the strength of these pTSSs was all upregulated under hypoxia (Table S2). These results suggest that Mtb and *M. marinum* exploit similar transcriptional regulation mechanisms to cope with hypoxic stress. In addition, although SigE was shown to be upregulated under hypoxic conditions [19] and 9 transcriptional units directly regulated by SigE after SDS exposure were defined using a DNA microarray [36], the global regulatory targets of SigE in hypoxic responses are still largely unclear. We identified 4 (*sigB*, *htpX*, *fxsA* and *clgR*) of 9 pTSSs previously defined as SigE regulatory units, which illustrated the reliability of transcriptional regulation characterization by Cappable-seq. Moreover, we identified 59 SigE-dependent TSSs, which broadened the knowledge related to the regulatory role of SigE in hypoxic responses.

Leaderless transcripts have been identified in different bacterial species. In our study, we found that over 38% of pTSSs initiate leaderless transcripts in *M. marinum*, higher than the frequencies in Mtb and *M. smegmatis* (approximately 25%) [22, 23, 26]. Further studies are needed to elucidate the roles of these leaderless transcripts and the differences of *M. marinum* with Mtb and *M. smegmatis*. Moreover, we found that transcripts with 5′ UTR of 4 nt or longer would not be efficiently translated (Fig. 3c); the role of this phenomenon would be worthy of further study. A recent study tried to elucidate the impact of leaderless transcript structures on translation efficiency, transcript stability, and transcription rates in *M. smegmatis* [47], but the roles of leaderless transcripts remain to be further investigated.

In summary, our study globally mapped the transcriptional regulation of *M. marinum* in response to hypoxia, providing valuable information for future studies of how Mtb transits into dormancy and potential new approach for global transcriptional investigations.

## Materials and methods

### Bacterial strains and growth conditions

The bacterial strains and plasmids used in this study are listed in Table S4 and oligonucleotides are summarized in Table S5. *M. marinum* cells were grown in Middlebrook 7H9 broth (Difco) supplemented with 0.5% (V/V) glycerol, 0.05% (W/V) Tween-80 and 10% (V/V) OADC (Difco) at 32 °C, 100 rpm or on Middlebrook 7H10 agar (Difco) supplemented with 0.5% (V/V) glycerol and 10% (V/V) OADC (Difco). *M. smegmatis* used for the eGFP reporter assay was cultured in 7H9 broth supplemented with 0.2% (V/V) glycerol, 0.05% (W/V) Tween-80, 0.2% (W/V) glucose and 15 mM NaCl at 37 °C, 200 rpm or on 7H10 agar plates supplemented with 0.5% glycerol. *E. coli* DH5α used for cloning was routinely grown at 37 °C in Luria-Bertani (LB) broth or on LB agar plates. The concentration of kanamycin used was 20 μg/ml for mycobacteria and 50 μg/ml for *E. coli* DH5α.

### Hypoxic treatment of *M. marinum*

The Wayne model [6] was applied for hypoxic treatment. Briefly, *M. marinum* and its derivative strains were aerobically grown to mid-exponential phase (OD_600_ = 0.6), and then diluted into fresh media containing 1.5 μg/ml methylene blue to a starting OD_600_ of 0.05. A volume of 24 ml diluted culture was transferred into a 36 ml screw-cap tube (2.5 cm × 8 cm). The solid cap with a latex liner was tightly screwed down and sealed with paraffin wax. After 24 h of incubation, the methylene blue had become completely decolorized, and the OD_600_ had reached 0.3. For the normoxic control, 96 ml of the same diluted culture was aerobically incubated in a 250 ml conical flask. Samples were also collected when the bacterial OD_600_ reached 0.3.

### *M. marinum* survival under hypoxic stress

*M. marinum* cultures prior to and after 24 h of hypoxic incubation were collected and serially diluted 10-fold. Specifically, 50 μl samples were diluted with 450 μl PBST_80_ (PBS supplemented with 0.05% Tween-80). Then, 100 μl aliquots of 10^− 4^ or 10^− 5^ dilution were spread onto 7H10 plates in triplicate. After 7 days of incubation at 32 °C, the number of colonies was counted. These tests were independently performed in triplicate.

### RNA extraction and qRT-PCR

RNA extraction and qRT-PCR were performed as described previously [15, 40]. Briefly, four copies of 24 ml of *M. marinum* subjected to hypoxic treatment or 96 ml *M. marinum* growing under normoxic conditions were harvested by centrifugation at 7500 rpm for 10 min at 4 °C and then frozen immediately in liquid nitrogen. After grinding in liquid nitrogen, the samples were transferred to 2 ml tubes containing 1 ml TRIzol (Invitrogen). RNA was extracted according to the manufacturer’s protocol. For qRT-PCR experiments, 2 μg of RNA was digested with RNase-free DNase I (Promega) and then reverse transcribed to cDNA using M-MLV reverse transcriptase (Promega). iTaq Universal SYBR Green Supermix (Bio-Rad) was used to perform qRT-PCR reactions in duplicate. The relative RNA levels of the tested genes were normalized to the levels of *sigA*. The mean values and standard error of mean (SEM) from biological triplicates are shown. Data comparisons between two groups were performed using Student’s t-test.

### Construction of regular RNA-seq libraries and sequencing

Regular RNA-seq was performed as previously described [48]. The rRNA in total RNA extracted from triplicate normoxic and hypoxic cultures was removed by a Ribo-off rRNA Depletion Kit (Vazyme), and libraries were constructed using the NEBNext Ultra Directional RNA Library Prep Kit for Illumina (NEB). Sequencing was performed on the Illumina HiSeq X Ten platform using 2 × 150 bp paired-end sequencing. Fold changes were calculated by comparing the fragments per kilobase per million mapped fragments (FPKM) between two conditions.

### Construction of Cappable-seq libraries and sequencing

RNA samples from triplicate normoxic and hypoxic cultures were subjected to enrichment for primary transcripts as previously described [21], with minor modifications. First, 1.8× Agencourt AMPure XP beads (Beckman) were used to purify RNA in all procedures. Second, to obtain enough primary transcripts for subsequent library construction, enrichment procedure using hydrophilic streptavidin magnetic beads (NEB) was performed for one time. The NEBNext Small RNA Library Prep Set for Illumina (NEB) was used to generate Cappable-seq libraries. To reduce the concentration of adaptor dimer, the 3′ SR adaptor and 5′ SR adaptor were both used at 4-fold dilutions. After 21 cycles of PCR amplification, the libraries were purified using a QIAquick PCR Purification Kit (QIAGEN) and 1.5× Agencourt AMPure XP beads (Beckman). The concentration and size distribution of the libraries were determined by Qubit 3 (Invitrogen) and Bioanalyzer DNA 1000 (Agilent) respectively. Sequencing was performed by the Illumina HiSeq X Ten platform.

### Identification and classification of TSSs

Clean reads of Cappable-seq were mapped to the *M. marinum* strain M ATCC BAA-535A genome (NC_010612.1 and NC_010604.1). The first nucleotides at the 5′ ends of the mapped reads were extracted as candidate TSSs. Candidate TSSs located within genes encoding rRNA or tRNA were excluded from further analyses. Based on the number of reads mapped to the subsequent 50 bp, the strength of candidate TSSs was calculated as the relative read score (RRS) according to a previous description [21]. Briefly, RRS was calculated as (Rns/Rt) × 10^6^, with Rns being the number of trimmed reads mapped to position n in the *M. marinum* genome on either strand (− or +) and Rt being the total number of reads mapped to the *M. marinum* genome. TSSs were classified into five categories according to genome position and TSS strength as previously described [20]. A TSS was classified as a primary TSS when it was located within ≤500 bp upstream of the annotated ORF. A secondary TSS was associated with the same ORF but had a lower RRS than the primary TSS. Internal TSSs assigned to TSSs were located inside an ORF on the same strand, and antisense TSSs were also located inside an ORF but on the opposite strand. A TSS was classified as an orphan TSS when it could not be assigned to any of the above four categories.

### Promoter motif search

To analyze the promoter motifs, 12 bp (− 5 to − 16 relative to TSS) and 16 bp (− 25 to − 40 relative to TSS) sequences of promoters were extracted to search for − 10 and − 35 motifs, respectively, using MEME version 5.3.0 (http://meme-suite.org/tools/meme) online [30, 31].

### Construction of plasmids

The eGFP reporter plasmids were constructed by inserting a fragment containing a synthetic promoter and *egfp* gene into the integrative plasmid pMV306 [38]. Different lengths of 5′ UTRs were introduced using the QuikChange II XL site-directed mutagenesis kit (Stratagene). Two aspects are considered for the introduced 5′ UTR sequences. First, the introduction of these sequences would not create potential ribosomal binding sites. Second, the introduction of these sequences would not change the secondary structure of *egfp* mRNA. Fragments of the *fadA5* gene were amplified from the genomic DNA of *M. marinum* strain M ATCC BAA-535A. PCR fragments were inserted into the linearized promoterless vector pMV306-*egfp* using a ClonExpress II One Step Cloning Kit (Vazyme).

### eGFP reporter assay

The eGFP reporter plasmids were transformed into *M. smegmatis*. Transformants from 7H10 agar plates were grown to an OD_600_ of 0.8 in 7H9 medium, harvested and resuspended in PBST. Cells were transferred to black 96-well plates to test the fluorescence intensities using Bio-TEK Synergy HT (Bio TEK) as described previously [33]. The eGFP expression levels are indicated by relative fluorescence units (RFU, fluorescence intensities per OD_600_). Two transformants in each group were tested in duplicate. Experiments were independently performed twice.

### Comparison of Cappable-seq and regular RNA-seq data

To test the efficiency of Cappable-seq in characterizing transcriptional regulation, pTSSs expressed under both normoxic and hypoxic conditions were chosen, and then the expression of genes downstream of these pTSSs was determined in regular RNA-seq. For comparison of the fold-changes of RNA levels with pTSS strengths, GraphPad Prism 8.0.2 was used to compute the nonparametric Spearman correlation.

### Statistical analyses of regular RNA-seq and Cappable-seq data

For multiple testing correction, FDR-adjusted *p*-values were obtained using DESeq2 [49] implemented in R (version 3.2.2). Genes or TSSs were considered to be differentially expressed at fold change ≥2 and adjusted *p* < 0.05.

## Supplementary Information

**Additional file 1.**

**Additional file 2.**

**Additional file 3.**

**Additional file 4.**

**Additional file 5.**

## Data Availability

The RAW data for Cappable-seq assays are available in SRA: PRJNA684793. The RAW data for regular RNA-seq assays are available in SRA: PRJNA684796.
