# Supplementary material for: Genome-scale analyses of transcriptional start sites in Mycobacterium marinum under normoxic and hypoxic conditions
Source: BMC Genomics. 2021 Apr 6;22:235. doi: 10.1186/s12864-021-07572-8 (PMC8022548; doi:10.1186/s12864-021-07572-8)
Supplement: Supplementary file 4 — Additional file 4. [file 12864_2021_7572_MOESM4_ESM.docx]

Table S4. Bacterial strains and plasmids used in this study.

| **Name** | **Descriptions** | **Sources** |
| --- | --- | --- |
| **Strains** |  |  |
| *M. marinum* | *Mycobacterium marinum* strain M ATCC BAA-535 | Dr. Jun Liu |
| *M. smegmatis* | *Mycobacterium smegmatis* mc^2^155 | Dr. Jiaoyu Deng |
| *E. coli* DH5α | Strain for cloning constructions | Lab collection |
| **Plasmids** |  |  |
| pMV306 | Integrative plasmid for mycobacterial strains, Kan^R^ | Stover *et al*., 1991 |
| pMV306-nop-eGFP | pMV306 carrying *egfp* gene without promoter, Kan^R^ | This study |
| pMV306-eGFP-UTR0~UTR12 | pMV306 carrying *egfp* gene with a synthetic promoter and different length of 5´ UTR, from 0 nt to 12 nt, Kan^R^ | This study |
| pMV306-*fadA5*p-eGFP | pMV306 carrying *fadA5*p upstream of *egfp* gene, Kan^R^ | This study |
| pMV306-*fadA5*p-*fadA5*-eGFP | pMV306 carrying *fadA5*p and *fadA5* translational fusion to *egfp* gene, Kan^R^ | This study |
