# Supplementary material for: Genome-scale analyses of transcriptional start sites in Mycobacterium marinum under normoxic and hypoxic conditions
Source: BMC Genomics. 2021 Apr 6;22:235. doi: 10.1186/s12864-021-07572-8 (PMC8022548; doi:10.1186/s12864-021-07572-8)
Supplement: Supplementary file 5 — Additional file 5. [file 12864_2021_7572_MOESM5_ESM.docx]

Table S5. Oligonucleotides used in this study.

| **Name** | **Sequences 5’→3’** | **Application** |
| --- | --- | --- |
| sigA-F | ggtccgcgcctacctcaa | qRT-PCR |
| sigA-R | gcggtctgtcaactcagccat |  |
| dosR-F | gcatcgaactgtgccgtga |  |
| dosR-R | aacatatccgctggctccc |  |
| tgs1-F | accgcagcagggttcgt |  |
| tgs1-R | cgccgagacccgattgt |  |
| inhA-F | gtctccaagggcatccaca |  |
| inhA-R | caaccgtcatccagttataggc |  |
| eGFP-F2 | acgtaaacggccacaagttc |  |
| eGFP-R2 | aagtcgtgctgcttcatgtg |  |
| pMV306-Kan-eGFP-F | aagaaggagatatacatatggtgagcaagggcgaggag | pMV306-eGFP fusions plasmids constructions |
| pMV306-GS-eGFP-F | ggtagtggttcaggttcgatggtgagcaagggcgaggag |  |
| pZT102-R1 | ggccgtgacgtgactagtaaaaaaggg |  |
| Fad5Ap-F | ctagtcacgtcacggccgaatccctcttgcggggc |  |
| Fad5Ap-R | atgtatatctccttcttatgtggtcaggctagaacacgtttc |  |
| Fad5A-R | aacctgaaccactaccgattcgctcgagaatggtgccg |  |
| 5UTR-F | atggtgagcaagggcgaggag | pMV306-eGFP-UTR0~UTR12 plasmids constructions |
| 5UTR-0-R | ctcgcccttgctcaccatgccgagatgatattggataatgcaacc |  |
| 5UTR-1-R | ctcgcccttgctcaccattgccgagatgatattggataatgcaacc |  |
| 5UTR-2-R | ctcgcccttgctcaccatatgccgagatgatattggataatgcaacc |  |
| 5UTR-3-R | ctcgcccttgctcaccatgatgccgagatgatattggataatgcaacc |  |
| 5UTR-4-R | ctcgcccttgctcaccatggatgccgagatgatattggataatgcaacc |  |
| 5UTR-5-R | ctcgcccttgctcaccattggatgccgagatgatattggataatgcaacc |  |
| 5UTR-6-R | ctcgcccttgctcaccatgtggatgccgagatgatattggataatgcaacc |  |
| 5UTR-7-R | ctcgcccttgctcaccatagtggatgccgagatgatattggataatgcaacc |  |
| 5UTR-8-R | ctcgcccttgctcaccattagtggatgccgagatgatattggataatgcaacc |  |
| 5UTR-9-R | ctcgcccttgctcaccatctagtggatgccgagatgatattggataatgcaacc |  |
| 5UTR-10-R | ctcgcccttgctcaccatgctagtggatgccgagatgatattggataatgcaacc |  |
| 5UTR-11-R | ctcgcccttgctcaccattgctagtggatgccgagatgatattggataatgcaacc |  |
| 5UTR-12-R | ctcgcccttgctcaccatgtgctagtggatgccgagatgatattggataatgcaacc |  |
